# Patient and clinician experiences with the implementation of telemedicine and related adaptations in office-based buprenorphine treatment during the COVID-19 pandemic: a qualitative study

**DOI:** 10.1186/s13722-025-00536-3

**Published:** 2025-03-04

**Authors:** Melissa Davoust, Angela R. Bazzi, Samantha Blakemore, Juliana Blodgett, Anna Cheng, Sarah Fielman, Kara M. Magane, Jacqueline Theisen, Richard Saitz, Alicia S. Ventura, Zoe M. Weinstein

**Affiliations:** 1 Department of Health Law, Policy, and Management, Boston University School of Public Health, 715 Albany St, Boston, MA 02118 USA; 2 Department of Community Health Sciences, Boston University School of Public Health, 715 Albany St, Boston, MA 02118 USA; 3 Herbert Wertheim School of Public Health, University of California San Diego, 9500 Gilman Drive, La Jolla, CA 92093 USA; 4 Grayken Center for Addiction, Clinical Addiction Research and Education Unit, Section of General Internal Medicine, Department of Medicine, Boston Medical Center, 72 East Concord St, Boston, MA 02118 USA; 5 Boston University Chobanian & Avedisian School of Medicine, 72 East Concord St, Boston, MA 02118 USA

**Keywords:** Telehealth, Telemedicine, COVID-19, Opioid use disorder, Medications for opioid use disorder, Buprenorphine, Qualitative research

## Abstract

**Background:**

Deaths from opioid overdose have increased dramatically in the past decade. For individuals with opioid use disorder (OUD), agonist medications such as methadone and buprenorphine reduce opioid-related morbidity and mortality. Historically, the provision of buprenorphine treatment in office-based settings has relied on frequent in-person contact, likely influencing patients’ access to and retention in care. In response to the COVID-19 pandemic, providers of office-based buprenorphine treatment rapidly adapted their care processes, increasingly relying on telemedicine visits. To date, relatively few prior studies have combined patient and clinician perspectives to examine the implementation of telemedicine and related care adaptations, particularly in safety-net settings.

**Methods:**

Qualitative methods were used to explore clinician and patient experiences with telemedicine in an office-based buprenorphine treatment clinic affiliated with an urban safety-net hospital. From this clinic, we conducted semi-structured interviews with 25 patients and 16 clinicians (including prescribers and non-prescribers). We coded all interview data and used a thematic analysis approach to understand how telemedicine impacted treatment quality and engagement in care, as well as preferences for using telemedicine moving forward.

**Results:**

Five themes regarding the implementation of telemedicine and other COVID-19-related care adaptations arose from patient and clinician perspectives: (1) telemedicine integration precipitated openness to more flexibility in care practices, (2) concerns regarding telemedicine-related adaptations centered around safety and accountability, (3) telemedicine encounters required rapport and trust between patients and clinicians to facilitate open communication, (4) safety-net patient populations experienced unique challenges when using telemedicine, particularly in terms of the technology required and the need for privacy, and (5) there is an important role for telemedicine in office-based buprenorphine treatment moving forward, primarily through its use in hybrid models of care which integrate both in-person and virtual visits.

**Conclusions:**

Telemedicine implementation within office-based buprenorphine treatment has the potential to improve patients’ engagement in care; however, our findings emphasize the need for tailored approaches to implementing telemedicine in office-based buprenorphine treatment, particularly within safety-net settings. Overall, this study supports the maintenance of changes to policy and practice that facilitate the use of telemedicine in office-based buprenorphine treatment beyond the COVID-19 public health emergency.

**Supplementary Information:**

The online version contains supplementary material available at 10.1186/s13722-025-00536-3.

## Background

Opioid overdose mortality has risen dramatically in the United States in recent years, with over 80,000 deaths reported in 2022 alone [1, 2]. Medications for opioid use disorder (MOUD) reduce morbidity and mortality, yet only an estimated 18% of U.S. residents with opioid use disorder (OUD) received MOUD in 2023 [3, 4]. Of those initiating treatment, many are not retained in care long-term [5].

Historically, models of providing MOUD, including office-based treatment with buprenorphine, have required frequent in-person contact [6]. Calls for low-threshold, patient-centered treatment approaches identify potential barriers to long-term engagement within these models, including their requirements for in-person evaluations prior to medication induction, regular urine toxicology testing, and short prescription intervals with refills tied to frequent in-person visits [6, 7].

The use of telemedicine for office-based buprenorphine treatment prior to the coronavirus disease (COVID-19) pandemic was limited [8, 9]. Beginning in March 2020, the COVID-19-related emergency response resulted in a rapid transformation of office-based buprenorphine treatment provision, most notably through the temporary removal or relaxation of legislative and regulatory barriers that enabled increased use of telemedicine [10]. For the first time, the Drug Enforcement Administration (DEA) authorized buprenorphine prescribing to new and existing patients with OUD via telemedicine without requiring in-person evaluations [11]. In addition, the Centers for Medicare and Medicaid Services (CMS) revised regulations to allow for reimbursement of audio-only telemedicine encounters, which increased flexibility by not requiring patients to use video [12]. Due to the need for pandemic-related social distancing, and facilitated by the aforementioned policy changes, providers of office-based buprenorphine treatment rapidly transitioned to telemedicine encounters [13, 14] and other changes in their care processes, including longer prescription intervals and reduced (or eliminated) urine toxicology testing [15].

Prior studies with clinicians engaged in office-based buprenorphine treatment have found telemedicine may help reduce barriers to treatment initiation and ongoing engagement in care, but also identified challenges including the potential loss of information gained through clinical and physical assessments, and patients’ limited technology access, particularly for video encounters [16–20]. Research with patients has indicated that telemedicine provided increased flexibility and autonomy, but also highlighted difficulties accessing medications and raised questions surrounding how telemedicine could differentially impact structurally marginalized populations [16, 21–27].

Relatively few prior studies have examined both patients’ and clinicians’ experiences with the implementation of telemedicine and related care adaptations within the same clinical contexts [23, 26, 27], and to our knowledge, none have been conducted within safety-net settings, or those that provide health services to patients regardless of their ability to pay. To expand the literature in this area, we conducted qualitative interviews with patients and clinicians in an Office-Based Addiction Treatment (OBAT) clinic affiliated with a large urban academic safety-net hospital to explore experiences with the use of telemedicine in this context, perspectives on how telemedicine can impact treatment quality and engagement in care, and preferences for the use of telemedicine moving forward.

## Methods

### Study setting

**Fig. 1:**
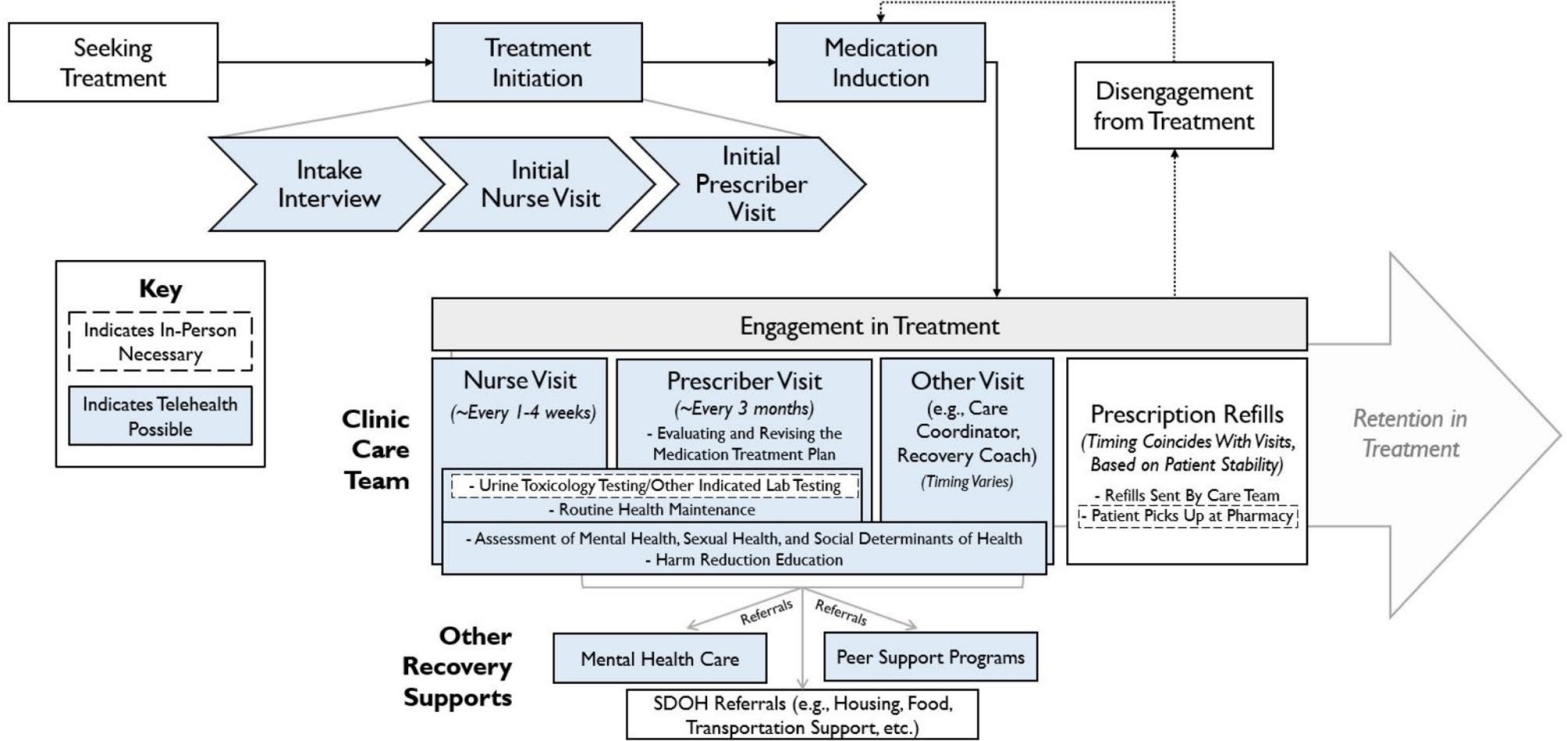
Nurse care manager model of office-based addiction treatment (OBAT)

From May 2021 to May 2022, we collected qualitative data as part of a larger cohort study examining the impacts of the COVID-19 pandemic in an OBAT clinic integrating buprenorphine treatment with primary care for patients with OUD [28]. Key features of the clinic’s model of care include: (1) high-touch patient contact, traditionally via in-person visits, and (2) the use of nurse care managers, who share clinical management responsibilities with prescribers and coordinate support from medical assistants, administrative staff, and other OBAT team members [28] (Fig. 1).

In March 2020, following the initial COVID-19 pandemic surge in hospitalizations, the clinic transitioned almost all patients to exclusively telemedicine encounters (both audio-only and video visits). During this time, all patients without access to a phone were given one by the clinic to minimize the need for in-person visits. However, the clinic continued in-person visits for patients requiring medication injections administered by clinical staff, as well as offered limited in-person visits for patients who needed them. In February 2021, the clinic shifted to a hybrid model blending telemedicine and in-person encounters, which continued throughout our data collection period.

### Study design and sample

To explore patients’ perspectives, we recruited a sub-sample of participants from a prospective cohort comprised of individuals who were at least 18 years of age, had a confirmed OUD diagnosis, had previously been prescribed any form of buprenorphine, and had at least one encounter with the OBAT clinic since January 2020. We used baseline survey data to purposively sample [29] patients with diverse socio-demographics (e.g., age, gender identity, and race/ethnicity) and experiences with the clinic who expressed willingness to participate in semi-structured qualitative interviews. Purposive sampling is used in qualitative research to select participants based on characteristics relevant to the goals of the study, and in this case we sought to include patients with a variety of backgrounds and perspectives [29]. To explore clinicians’ perspectives, we purposively sampled personnel with a range of roles in the OBAT clinic whose employment began at least three months prior to the onset of the COVID-19 pandemic; study team members worked within their professional networks to contact potentially eligible clinicians to inform them about the study. Recruitment communications were primarily conducted remotely (e.g., by phone or secure email). From all eligible individuals (patients and clinicians) who expressed interest in participating, trained interviewers elicited verbal informed consent. Participants received a $50 debit card for completing qualitative interviews. The Boston University Medical Campus Institutional Review Board reviewed and approved all study protocols, including a waiver of documentation of consent.

### Data collection

Participants completed one-time qualitative interviews by phone or video conference. Trained interviewers used semi-structured guides tailored to patients or clinicians that contained open-ended questions and optional, detailed probes to elicit information on key domains of interest (e.g., experiences before and during the pandemic, perspectives on telemedicine and other key changes in care processes, suggestions and preferences moving forward) [30]. For example, clinicians were asked questions such as “What are the most important ways that COVID-19 impacted the clinical services provided through the OBAT program?” and patients were asked questions such as “Reflecting on your experience with OBAT during the COVID-19 pandemic, what suggestions would you have for improving the care/services you’re receiving?”. The full interview guides can be seen in the Supplemental Materials. Interviews lasted between 23 and 56 min (clinicians) and 26–80 min (patients) and were recorded for professional transcription. Study personnel reviewed transcripts for quality and to ensure de-identification following a structured protocol (see Supplemental Materials) [31]. Audio recordings were destroyed after quality control processes were completed.

### Data analysis

The initial stages of data analysis included two key steps: (1) team-based coding, and (2) use of the framework method [32]. First, the team-based coding process was completed separately for each dataset (i.e., patients and clinicians), starting with collaborative codebook development [33, 34]. Two lead qualitative investigators developed initial lists of deductive codes based on overarching study questions and emergent topics documented in interviewer and team meeting notes. Study team members (including the lead qualitative investigators and research assistants) independently read selected transcript excerpts and applied the codes, writing memos on their overall experiences and areas for potential codebook improvements. Through regular meetings, the team discussed experiences with code application, resolved coding discrepancies, and revised the codebook for testing on new sets of transcript excerpts. We repeated this process until reaching consensus on the final codebooks for patients and clinicians, which included codes such as “In-Person Visits”, “Telehealth Visits”, “Technology”, “Environment”, “Patient-Clinician Interactions/Relationships”, “Urine Toxicology”, and “Medication Access”, among others. The final codebooks can be seen in the Supplemental Materials.

Once the codebooks were finalized, they were uploaded along with full interview transcripts into NVivo (Release 1) for code application. Members of the study team double-coded approximately 2–3 full transcripts per dataset using NVivo (Release 1), meeting regularly to resolve discrepancies. After establishing consistency, the team proceeded to single code the remaining transcripts in each dataset, continuing to meet to discuss coding progress and emergent findings.

Next, once coding was completed, we further organized data according to the framework method [32]. This method represents a helpful interim step in the process of qualitative analysis that provides a structured approach for managing and synthesizing qualitative data. The framework method refers to a process of distilling large quantities of qualitative data in a matrix that is organized conceptually (i.e., by code) while still ensuring views from each participant remain connected (i.e., by case). The study team members who had completed coding for each dataset reviewed and summarized data within each code by participant using a framework matrix (i.e., spreadsheet). In addition to brief summaries, they selected representative quotes to support the summaries and wrote higher-level memos interpreting data across participants by code. Throughout the process of developing the framework matrix, the study team continued to meet weekly to discuss progress, team members’ interpretations of the data, and potential emergent sub-themes. The framework method was chosen as an interim step in analysis as it can be useful when working in research teams, as well as when working with heterogenous data, or that which covers multiple topics and key issues, both of which were true for this study.

**Fig. 2:**
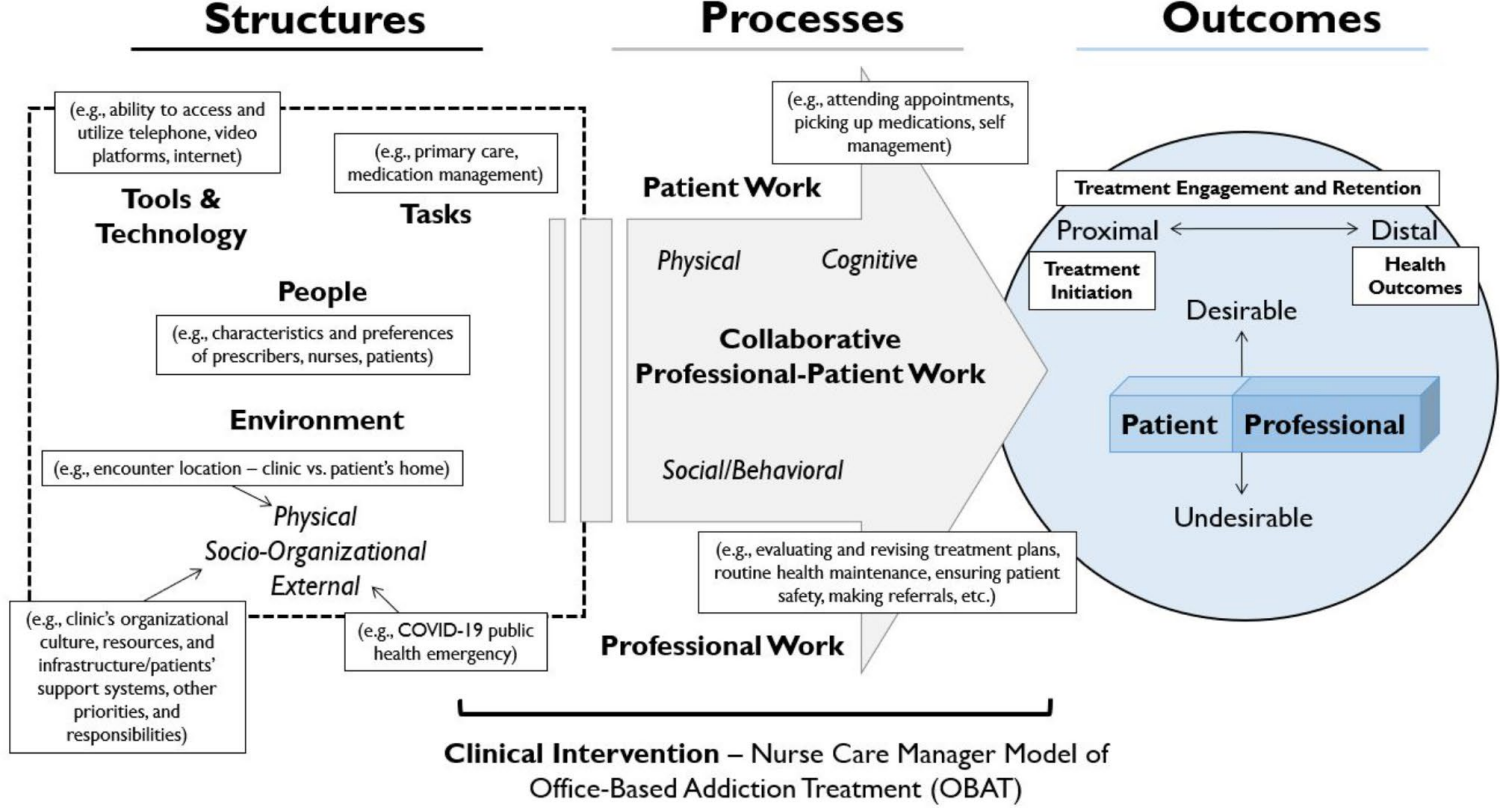
Conceptual framework for examining patient and clinician experiences with the implementation of telemedicine and related adaptations (Adapted SEIPS Model)

The framework matrix, along with all memos compiled in the preceding process (e.g., during recurring meetings), informed a thematic analysis in which a lead qualitative investigator (MD) synthesized the higher-level interpretations of the data in the framework matrices within and across datasets (i.e., patients and clinicians) [35, 36]. The thematic analysis was primarily a theoretical (or deductive) approach [36] guided by an adapted version of the Systems Engineering Initiative for Patient Safety (SEIPS) Model (Fig. 2) [37, 38], and most themes identified were at the latent (or interpretive) level, in that they represent underlying ideas and conceptualizations beyond what was explicitly stated by participants [36]. Findings are illustrated in the sections below using representative quotes.

## Results

### Sample characteristics and overview of key findings

Our final sample consisted of 41 participants, of which 25 were patients and 16 were clinic staff. Socio-demographic characteristics of the patients interviewed were similar to the broader population of the clinic (Table 1), which is majority White, Non-Hispanic male patients with an average age in the mid-40s. The staff interviewed included mix of seven prescribing (e.g., physicians [MD] and nurse practitioners [NP]) and nine non-prescribing (e.g., nurse care managers and program staff) care team members. From these qualitative interviews, we identified five themes shared among patients and clinicians regarding the implementation of telemedicine and other COVID-19-related adaptations: (1) telemedicine integration precipitated openness to more flexibility in care practices, (2) concerns arose regarding safety and accountability following telemedicine-related adaptations, (3) telemedicine encounters required rapport and trust between patients and clinicians to facilitate open communication, (4) safety-net patient populations experienced unique challenges when using telemedicine, particularly in terms of the technology required and need for privacy, and (5) telemedicine could play an important role in office-based buprenorphine treatment moving forward, primarily through its use in hybrid models of care.

**Table 1:** Characteristics of patient interview participants (*n* = 25)

	*n* (%)
Age (Mean, SD)	49 (11)
Age Range	32–67
*Gender Identity*	
Male	16 (64%)
Female	9 (36%)
English as Primary Language	24 (96%)
*Race/Ethnicity*	
Black, Non-Hispanic	7 (28%)
White, Non-Hispanic	16 (64%)
Other	2 (8%)
*Housing Status*	
Unstable (e.g. street/outdoors, shelter, etc.)	4 (16%)
Stable (e.g. own/rent, supportive housing, etc.)	21 (84%)
*Education Level*	
Elementary-High School	16 (64%)
College/Technical School	9 (36%)
*Employment Status*	
Employed/Student	13 (52%)
Unemployed, Disabled, or Retired	12 (48%)
*Insurance Status*	
Medicaid	19 (76%)
Medicare	4 (16%)
Private	2 (8%)
*Prescribed Medication for Opioid Use Disorder*	
Sublingual Buprenorphine	22 (88%)
Injectable Buprenorphine	3 (12%)

### Telemedicine integration precipitated openness to more flexibility in care practices

Participants reflected on their experiences with the clinic’s model of care prior to the pandemic, during its height, and the blend of telemedicine and in-person care being used at the time of the interviews. Clinicians described the adaptations to care processes precipitated by COVID-19 as having provided them with a new perspective.

*“I think the OBAT program has traditionally been very systemized… People come in*, *they get their tox screens*, *they have their visits… I think we realized we could do things in different ways.” [Prescriber #1]*.

During the shift to telemedicine during the COVID-19 pandemic, clinicians became more comfortable with and open to flexibility in care practices, which they felt benefited their patients.

*“I definitely think that telemedicine should stay… as a piece of the entire spectrum of services. I believe we should see our patients in-person*, *but that telemedicine can be a supplement… and can augment the care plan… [to] keep people engaged in care.” [Non-Prescriber #1]*.

Though there was increased openness to changes in the model of care, participants also indicated the stage of office-based buprenorphine treatment (e.g., treatment initiation or ongoing care engagement) played an important role in terms of how flexible they felt care practices should be.

#### Treatment initiation

Clinicians felt telemedicine could facilitate low-barrier access to care, which greatly benefited their patients. They highlighted the importance of telemedicine for those that require urgent connection to care to reduce the risk of overdose.

*“I think we should keep [telemedicine] for people who are engaging from an acute treatment setting or from incarceration*, *people that need to continue or start on medication right away to decrease their risk of… overdose.” [Prescriber #2]*.

They also highlighted how telemedicine could expand access in the face of soaring need.

*“I think we’re in an epidemic of opiate use*, *so I don’t think there was ever a question of whether or not somebody was calling us and truly did or didn’t have substance use. I think it was a really huge relief for a lot of people who could not get connected to health [care]*, *to be able to get connected to health [care].” [Non-Prescriber #2]*.

Although clinicians felt that some initial tasks (e.g., taking patient history, reviewing risk factors for overdose) could be done via telemedicine, they also noted the importance of in-person physical exams and laboratory data for assessing co-morbid conditions and complications of substance use disorders (e.g., from injection drug use) that were difficult to assess via telemedicine. Therefore, many also emphasized the need for in-person visits at this stage of treatment.

Another factor related to the perceived appropriateness of telemedicine for treatment initiation arose from the patient perspective. Though they also discussed a variety of positive aspects of telemedicine, many patients emphasized the importance of in-person visits and elements related to an in-person model of care. They felt the appropriateness of telemedicine visits in office-based buprenorphine treatment was dependent on an individual’s stage of recovery. Patients discussed their views on this retrospectively–considering what they felt would have been appropriate when they were new to treatment and recovery–as well as when considering their preference for telemedicine or in-person visits at present and moving forward.

*“If you have a patient that’s at a place in their recovery where they are honest and they’re committed and they want this… [telemedicine is] a great way to maintain your recovery and get things done… But if I was in a different place in my recovery*, *I don’t know if the accountability would have been there at all.” [Patient #1]*.

A key factor related to whether patients considered telemedicine to be appropriate was the perceived need for structure and routine, particularly early in treatment. Some clinicians also felt in-person visits were important for patients who were in the process of stabilizing on medication, or for those who could benefit from the routine associated with frequent check-ins.

*“If someone was struggling*, *seeing them more often*, *we felt*, *was a protective factor*, *so seeing them once a week as opposed to seeing them once a month.” [Non-Prescriber #3]*.

#### Ongoing care engagement

Participants emphasized how having telemedicine as an option for visits reduced barriers to patients’ engagement in care, such as: (1) the need to travel, which was challenging for those who lived far away or had limited mobility; (2) financial costs (e.g., childcare or transportation); and (3) competing priorities, such as work, school, or caregiving.

*“The [clinic] stays open late a couple of nights a week*, *but still it’s a pain. Especially if you’re just trying to live your life… it feels like an anchor attached to you.” [Patient #2]*.

Other benefits of the flexibility of telemedicine included its use in emergencies when patients would otherwise have missed an appointment, and reducing concerns around COVID-19 exposure (e.g., using public transportation or waiting at the clinic). Clinicians felt that telemedicine allowed them to maintain some form of contact with patients when a strict in-person model may have otherwise led patients to disengage from care. They described how telemedicine could increase retention by facilitating an outreach-oriented approach to care.

*“The positive thing was being able to have [patients] engaged more often [but] not feeling like they’re handcuffed…to have to come in and provide urines and do this whole thing…[And] we were really responsive with them*, *so for example… If we didn’t get [ahold of] them*, *we would put them in our schedule to follow up.” [Non-Prescriber #2]*.

In some cases, patients described telemedicine as facilitating additional access to their clinicians.

*“You can use [telemedicine] more frequently*, *rather than specifically having to call and make an appointment with the doctor*, *or call and hope a nurse calls you back within the week… I’m able to reach out to my doctor[now]… It’s like having extra access to your physician*, *versus just having to book an appointment and wait for it.” [Patient #3]*.

Overall, participants varied in their individual preferences for the use of telemedicine, indicating the need for ongoing flexibility. Clinicians indicated decisions regarding the frequency of in-person versus telemedicine visits should depend on the needs and desires of individual patients, and most patients expressed a preference for combined in-person and telemedicine visits. When asked about their ideal balance of telemedicine and in-person care, patients’ individual preferences related to visit type and the clinician they would be seeing (e.g., in-person visits with prescribers, who were often primary care providers who participants wanted to see “face to face,” versus telemedicine for check-in appointments with nurses for buprenorphine prescription refills).

### Concerns regarding safety and accountability following telemedicine-related adaptations

Though there was recognition of the need for flexibility and related benefits that telemedicine could provide, participants also expressed concerns about related care adaptations associated with fewer in-person visits. The clinic’s previous model of care involved high-touch patient contact, with route urine toxicology testing at every nurse visit. From the clinician perspective, concerns around reducing or eliminating this practice centered on having adequate objective data to ensure patient safety. Some appreciated having urine toxicology results as a source of clinical information that could help facilitate open discussions with patients; as such, those clinicians preferred patients attend visits in-person so as to facilitate the collection of this data.

*“[Urine toxicology testing] gives me an opportunity to have a conversation*, *ask specific questions on how they’re doing with the recovery… Maybe they need a dose adjustment [or] extra support. Maybe I need to encourage them to get psychiatric care [for] anxiety or depression…So*, *it’s just gives me some more information that I can use to continue to support the patient.” [Prescriber #3]*.

However, other clinicians noted there was little evidence on the benefits of routine urine toxicology screening and felt that telemedicine could still enable high-touch contact without imposing unnecessary requirements on patients.

*“Pre-COVID*, *we had a firm structure of*, *you start with weekly visits*, *then after five to six visits – once your substance use disorder is stable – then you go to two weeks*, *then three weeks*, *then four. And your prescriptions for buprenorphine match that increment… For me*, *I have a huge amount of relief that we’re not urine tox screening people all the time*, *and that we’re extending our model to be able to increase frequency of touch without imposing.” [Non-Prescriber #4]*.

From the patient perspective, many valued certain programmatic elements of treatment, including the structure provided by having to attend appointments in-person regularly, having group or individual counseling sessions, and providing urine samples.

*“Just the commitment [of] having to go somewhere…That made a huge difference for me in the beginning of my recovery. Committing to even go to an appointment was huge… showing up was huge.” [Patient #4]*.

Patients felt that for those newer to recovery, these elements provided an important sense of “accountability.” The value placed on accountability related to patients’ desires for extrinsic motivation to avoid recurrence of substance use, as well as feelings of not “disappointing” clinicians.

*“I always went for my urines on a monthly basis…Then COVID-19 happened*, *and nobody could go in. [But] urine is what*, *you know*, *helps a lot… Even though that I’m not using [substances] or anything like that*, *it [is] just something to attach myself to.” [Patient #5]*.

For some, they saw it as most important when they were early in treatment, while others still appreciated the structure of regular in-person visits and testing though they were in longer-term recovery. A few patients did see urine toxicology testing as unnecessary and inconvenient, particularly those who had been in long-term treatment, and were more likely to endorse a desire for continued visits via telemedicine.

*“I never had any issues with [telemedicine visits]. I can’t speak for somebody who was still maybe relapsing often*, *or new to recovery*, *and maybe needed a little bit more hands-on of an approach… But for me*, *where I was so far along in recovery… I kind of benefited from almost*, *like*, *a hands-off approach.” [Patient #6]*.

### Telemedicine encounters required rapport and trust between patients and clinicians to facilitate open communication

As encounters changed from in-person to virtual, participants described how patient-clinician interactions and relationships were altered. In this patient population, most telemedicine visits were audio-only, which eliminated the visual connection between patients and clinicians. For clinicians, combined with the absence of other objective data sources (e.g., physical exams and urine toxicology results), this led to an increased reliance on positive rapport during interactions and building trusting relationships with patients. Establishing this connection and trust facilitated more open communication, which clinicians felt helped ensure they had the necessary subjective data to ensure patient safety (e.g., potential exposures to illicit substances that increased a patient’s risk of overdose).

*“For me*, *[transitioning to telemedicine has] taught me to communicate better… I don’t care what someone’s tox screen says*, *I’d rather build a relationship… Not having [urine toxicology results]*, *we can still take care of our patients… There are other tools that we can use [like] building relationships…and having the trust of our patients*, *and having to communicate our concerns…Those are tools that I didn’t use before [the pandemic] that I learned how to use.” [Non-Prescriber #2]*.

Patients also emphasized the importance of establishing rapport with clinicians. Many felt that visual connection played an important role (“*I would prefer in-person with the doctor*, *or worst-case*, *video”*). Though notably, many patients lacked the capacity or ability to use video during their telemedicine encounters.

Among both clinicians and patients, individual levels of comfort with telemedicine and establishing relationships via this modality varied. Some felt that in-person visits facilitated more open interactions, while others experienced more candid communication when using telemedicine.

*“Something that has bowled me over with [telemedicine] is…how different the interaction is if you can [get] away from the hospital… The level of intimacy that has been built by having patients be able to talk in a place that maybe feels more comfortable has been incredibly surprising.” [Non-Prescriber #4]*.

*“I’ve always felt that I’m more comfortable in my home. And I feel less rushed… I feel like it’s much more personal*, *I can open up and say the right things. And I don’t feel as out of sorts or rushed.” [Patient #6]*.

Among clinicians, some naturally felt more open to seeing both new and existing patients via telemedicine, while some preferred in-person encounters for both. Though one clinician did highlight the perceived importance of in-person encounters to establish trusting relationships with new patients.

*“What I think has changed is losing that face-to-face capability*, *and for patients that I’ve never met in person before*, *there is some slight hesitancy in telling me things about their use or their rituals or their recurrence of use*, *because they have no idea what I look like. I think a lot of times when you put a face behind the voice or a face behind the name*, *you enact more of a rapport and relationship.” [Prescriber #2]*.

Yet, this clinician also mentioned that certain patients may feel more comfortable sharing when not in person.

*“For some people it’s better because then they don’t have to tell someone in person. So*, *I would say that-that it’s kind of split*, *but I’ve seen more now wanting to [be seen] in-person and have that touch and that rapport.” [Prescriber #2]*.

Overall, participants emphasized how telemedicine encounters required a positive rapport and trusting relationship to facilitate open communication and information sharing. However, the degree to which individuals felt comfortable with telemedicine differed, and this level of comfort could affect the quality of clinical encounters. Therefore, most participants felt both options should be available to patients, depending on what works best for them.

### Safety-net patient populations experienced unique challenges when using telemedicine

Participants indicated that the use of telemedicine in a safety-net setting presented unique issues and that patients’ circumstances could differentially impact their experiences with telemedicine. The mechanisms underlying these differential impacts fell under two main categories: (1) the technology used in telemedicine encounters, and (2) the environment in which encounters took place.

Participants emphasized how seeing the other individual (e.g., faces, body language) during clinical encounters was important; however, many patients had unreliable access to the necessary technology, including smart phones or tablets and stable Internet connections, making video visits impossible.

*“I just did phone [visits]… In my halfway house*, *75% of the time we didn’t have access to the Internet ‘cause it was always down or there were too many people trying to get on it.” [Patient #7]*.

Some struggled with even having consistent access to a phone, particularly those with unstable housing. Technological literacy also played a role, particularly in terms of patients’ comfort with and ability to use video platforms.

*“It wasn’t Internet access [but] I guess you could say…I’m very simple*, *like technology and me don’t get along very well. [I spent a] third of my life in federal prison…when I went [in]*, *Nintendo was the big thing. I get out; they’ve got iPhones.” [Patient #8]*.

*“I have so many issues getting on the video… I don’t mind doing the phone call thing*, *[and] a lot of times I’d rather be in person*, *but [the] video thing [is] useless to me.” [Patient #3]*.

Participants also described the importance of patients’ surrounding environments during telemedicine encounters, noting the need for privacy to discuss sensitive health topics, and limited distractions. This was particularly difficult for patients living in congregate settings (e.g., homeless shelters, recovery housing) or with family (e.g., multigenerational households).

*“I live in a sober house*, *it’s not easy to find some place that’s quiet. But now I have a car*, *so I can hide in here…[addiction treatment is] not something you want to talk about in front of people.” [Patient #4]*.

*“My kids are here all the time; there’s no privacy. It’s a small*, *two-bedroom apartment. I try to not let them hear what I’m talking about.” [Patient #9]*.

Clinicians also expressed frustration with patients’ distractions during telemedicine visits (e.g., having appointments while driving or working), which they felt negatively affected interactions.

*“Patients don’t…give it the same attention as [they would if they] had to take a little bit of time off*, *come to the clinic*, *sit in a quiet private room…and have an actual conversation about what’s really going on.” [Prescriber #3]*.

In general, participants acknowledged that moving to telemedicine during the pandemic likely differentially impacted certain patients, including higher risk individuals with co-morbid mental health conditions or recurrence of substance use, who would have been more likely to walk into the clinic pre-pandemic.

*“My most vulnerable patients often don’t have a phone… I lost them all… [They] were extremely vulnerable and were just gone overnight*, *with no way to track them down.” [Non-Prescriber #4]*.

### Role of telemedicine in office-based buprenorphine treatment moving forward

Considering the aforementioned positives and negatives of integrating telemedicine in office-based buprenorphine treatment, participants agreed that there was a role for this new modality of care moving forward.

*“I think telehealth*, *as many challenges as there were*, *was a great place to meet patients where they’re at… It can be a lot to ask people to come into the clinic*, *but from a harm reduction standpoint*, *if patients have a stable way of communicating and we can get in touch with them*, *I think that’s a great way to streamline the process.” [Prescriber #2]*.

Patients varied significantly in terms of their individual preferences for engaging in care via in-person visits versus telemedicine, but overwhelmingly appreciated having the option of different modalities.

*“There’s pros and cons… It’s not always easy to get out to scheduled appointments [but] at the same time*, *it is good to see your health care provider face-to-face.” [Patient #1]*.

For example, some patients appreciated being able to have telemedicine encounters in different environments because they preferred avoiding the area around the clinic. Clinicians also identified this benefit, noting that some of their patients found the location triggering.

*“As far as a new patient not coming to an appointment*, *I think because of where our clinic [is]… There’s a lot of triggers around there… So*, *a lot of patients are like*, *‘I’m not comin’ in there. Because if I have to go down that street*, *it’s all over for me*, *I already know it.’” [Non-Prescriber #5]*.

Clinicians felt telemedicine should continue to be offered as an option for patients, particularly based on its potential benefits for ensuring retention in care; however, they also felt there should be consideration of clinical indication and stage of treatment. Though many felt in-person visits were still important, most clinicians saw telemedicine visits as being appropriate for long-term patients and supported telemedicine inductions to improve access to care.

*“I think intermixing some virtual appointments with in-person is fine to improve access [and] convenience for patients. I don’t think it’s always necessary for patients to be seen in person*, *but yet*, *we shouldn’t do without in-person visits*, *because I think there’s something important in seeing people in-person.” [Prescriber #4]*.

*“I would recommend keeping telemedicine access for whom it seems to be clinically appropriate*, *including the easing of regular urine drug screening [for] folks who are stable… I think the ongoing access and flexibility that telemedicine offers… will enable continued longer-term retention in care*, *especially for stable patients.” [Prescriber #5]*.

## Discussion

Reflecting on the changes made in response to the COVID-19 pandemic, patients and clinicians in this qualitative study agreed that their experiences with telemedicine for buprenorphine treatment were generally positive, particularly the increased flexibility it provided for patients. However, they also expressed some concerns about telemedicine-related adaptations, including the reduced use of urine toxicology testing, which some participants viewed as potentially impacting patient safety and feelings of accountability. Participants also commented on aspects of open communication, trust, and rapport between patients and clinicians, which could be difficult to cultivate in the absence of visual connections, which were not often possible in telemedicine encounters with this safety-net patient population. Both clinicians and patients emphasized that safety-net patient populations experienced unique challenges with telemedicine, including having the necessary technology and privacy to ensure quality encounters. Finally, many participants emphasized that while telemedicine could play an important role in office-based buprenorphine treatment moving forward, it was best to offer hybrid models of care, so patients had both in-person and telemedicine options available to them.

Our findings from the patient perspective also align with previous studies, which have found patients appreciated having options and the flexibility telemedicine provided in the context of competing demands and priorities in their lives [16]. However, though telemedicine may offer distinct benefits, particularly in helping patients overcome barriers to appointment attendance, a notable finding in this study was that patients’ views on the appropriateness of telemedicine visits centered around an individual’s stage of recovery, with the perceived need for structure and accountability early in treatment as a key concern. Previously, this was facilitated by regular in-person visits and routine urine toxicology testing, which have traditionally been perceived as appropriate and beneficial to some patients’ care [21]. However, it is worth noting that the desire for structure and accountability may be influenced in part by internalized stigma, and it is worth investigating if patients feel such practices could be eliminated or reduced if the elements they value (e.g., extrinsic motivation to avoid recurrence of use, feelings of trust with clinicians) can be provided by other means. In general, patient preferences around these elements of office-based buprenorphine treatment should be examined critically with regard to the history of punitive, monitoring-based approaches to addiction treatment. The acceptability of telemedicine as a modality among patients may be tied to historical attitudes toward addiction and addiction treatment, as well as patients’ own prior experiences within these structures of care.

Our findings from the clinician perspective align with prior work in which clinicians felt telemedicine had benefits such as increased access and convenience for patients [16]. Clinicians in this study felt there was a role for telemedicine in their practice moving forward, particularly to improve patients’ engagement and retention in treatment. Clinicians in prior studies have expressed some hesitancy to use telemedicine for office-based buprenorphine treatment generally [14], and for the initiation of treatment specifically [17]. Notably, clinicians in this study were generally comfortable with initiating treatment via telemedicine, citing its potential benefits in terms of low-barrier access to care. However, many did express a preference for seeing patients in-person within a certain period of time following medication induction, particularly for assessing co-morbid conditions, which is likely related to this being an integrated primary care model.

Finally, similar to previous studies [21, 24], our findings highlight the importance of patient-clinician interactions in ensuring high-quality clinical encounters via telemedicine. Participants emphasized how telemedicine visits required a positive rapport and trusting relationship to facilitate open communication and information sharing. However, an overarching finding was that individual clinicians and patients preferred different encounter modalities, and the degree to which individuals felt comfortable with telemedicine differed, indicating the importance of allowing options and not using one particular approach [21, 24]. In the historical context of MOUD treatment, offering telemedicine encounters as an option for patients based on their personal preferences represents progress toward more patient-centered care. However, this study also highlighted considerations specific to the implementation of telemedicine with safety-net patient populations.

Limited technology access and technological literacy were key issues impacting whether or not patients had video encounters–or virtual visits at all–and our participations emphasized that visual contact often provided important context during encounters. Although this clinic provided cell phones to patients who needed them, which has been shown to improve engagement [39], they were not smart phones and did not allow for video visits. Findings from this study suggest the modality of telemedicine–particularly the use of primarily audio-only encounters–may potentially affect patients’ experiences and perceived quality of care. Another issue that arose for this patient population was ensuring their physical environments allowed for private telemedicine appointments, which was particularly challenging for patients experiencing homelessness. In another study of how the COVID-19 pandemic impacted MOUD and addiction treatment access for individuals with OUD, Harris and colleagues found that some patients experienced telemedicine as “destabilizing” [24]. They suggested telemedicine may exacerbate inequities based on factors such as lack of housing and other social supports, which were important for ensuring patients could meaningfully engage in telemedicine encounters [24]. These social determinants similarly impacted the experiences of patients in our study. Taken together, these findings indicate the implementation of telemedicine can potentially differentially impact the most vulnerable patients receiving care in safety net settings.

Our findings should be considered in light of several limitations, particularly regarding generalizability. We recruited participants from a single office-based buprenorphine treatment clinic that previously relied on frequent in-person visits, meaning the perspectives of many clinicians and patients in our sample were influenced by prior experiences with this model of in-person care. Our findings are unlikely to generalize to experiences with telemedicine-only services; they also reflect an urban context in which some barriers to accessing care may be reduced compared to settings lacking any in-person care options (e.g., rural settings in which telemedicine is the only way to access buprenorphine treatment). Similarly, our study setting provided integrated primary care and addiction treatment, along with a variety of wraparound services and referrals [40]. Many patients in this study engaged with their prescriber for both OUD treatment and primary care, and some may have preferred to attend visits in-person for other reasons, such as accessing the available wraparound services. Despite these limitations to generalizability, our findings are likely still transferable to other safety-net settings and integrated primary care and office-based buprenorphine treatment programs [40, 41].

### Implications

Overall, our results support telemedicine as an acceptable method for providing office-based buprenorphine treatment for individuals with OUD and suggest it may improve access to and retention in care for many patients. These findings align with those from prior studies indicating that the COVID-19-related regulatory flexibilities around the use of telemedicine for office-based buprenorphine treatment should be maintained [16, 20]. In addition, patients in this study–the majority of whom were publicly-insured–expressed a desire to have a combination of telemedicine and in-person visits moving forward. CMS should consider the importance of parity in reimbursement for telemedicine so clinicians can ensure decisions regarding encounter type are based on patient preference [42, 43].

In terms of clinical practice, this study highlights a few considerations for clinicians and health systems. In particular, our findings highlight a need for tailored approaches to implementing telemedicine in office-based buprenorphine treatment, particularly within safety-net settings. First, proactive approaches to build rapport and trust can facilitate the collection of information from patients during telemedicine encounters that was previously garnered through in-person visits; therefore, developing and testing training on such approaches may be beneficial when shifting to telemedicine and introducing related changes (e.g., eliminating routine urine toxicology testing) to ensure clinicians’ concerns around patient safety are alleviated. Second, while telemedicine has its benefits, clinicians caring for safety-net patient populations should ensure in-person visits are still available, as moving to exclusively telemedicine-based approaches may systematically exclude already marginalized populations. Supports should also be provided to reduce disparities in patients’ access to telemedicine and their ability to use it for high-quality encounters. At minimum, this could include distributing cell phones for audio-only visits; however, particular attention should be paid to finding ways to support safety-net settings in their ability to provide additional technological and educational resources to ensure all patients are similarly equipped to utilize telemedicine when they choose to do so, particularly videoconferencing.

## Conclusions

The use of telemedicine in office-based buprenorphine treatment has the potential to improve patients’ engagement and retention in care. However, findings from this study emphasize the importance of hybrid models of care, and not using a one-size-fits all approach when implementing telemedicine in office-based buprenorphine treatment, particularly in safety-net settings. Both patients and clinicians emphasized the importance of patient preference in determining the frequency and modality of visits. Moving forward, telemedicine represents an important tool that can be used in the context of office-based buprenorphine treatment to shift from standardized models of addiction treatment with little flexibility to those that emphasize a patient-centered approach.

## Electronic supplementary material

Below is the link to the electronic supplementary material.

Supplementary Material 1

## Data Availability

The data and materials used for this study are available upon reasonable request to the corresponding author.

## Abbreviations

CMS: Centers for Medicare and Medicaid Services
COVID-19: Coronavirus Disease
DEA: Drug Enforcement Administration
MOUD: Medications for Opioid Use Disorder
OBAT: Office-Based Addiction Treatment
OUD: Opioid Use Disorder
