# Supplementary material for: Patient and clinician experiences with the implementation of telemedicine and related adaptations in office-based buprenorphine treatment during the COVID-19 pandemic: a qualitative study
Source: Addict Sci Clin Pract. 2025 Mar 4;20:21. doi: 10.1186/s13722-025-00536-3 (PMC11877703; doi:10.1186/s13722-025-00536-3)
Supplement: Supplementary file 1 — Supplementary Material 1 [file 13722_2025_536_MOESM1_ESM.pdf]

Transcript Quality Control (QC) & De-Identification Protocol

1. Access the “Interview Transcripts” folder.
2. Copy the transcript you are planning to QC (to ensure you do not edit the original document).
3. Ensure that the start of every transcript document lists the study name, Study ID, interview date, and interviewer initials. Remove extraneous information (e.g., file or transcriber names).
4. Ensure that formatting is consistent across all transcripts (use Arial font, size 11, single spaced, 1” margins, with page numbers in lower corner; remove all transcription company names and logos).
5. Find & replace “Interviewee” with the appropriate Study ID (in Word, Edit menu > Find & Replace)
6. Read through the entire transcript for:
    a. “Face validity:” Ensure that the content and terminology seems accurate (watch for potential errors a non-expert transcriptionist could have made with respect to jargon, slang, acronyms, medication or drug names).
    b. De-identification: Ensure that personally-identifying information has not been transcribed. In any cases where names, exact dates, or contact information was missed by the transcription company and remains in the transcript, please refer to the additional notes below.
    c. In-audible passages: Please flag any of these instances. If possible, we will have the interviewer listen to the audio recording to see if any additional information can be discerned (if they remain unsure, annotate this within brackets). Additional guidance:
        i. For in-audible passages you can confidently understand, go ahead and add it into transcript, then delete the “inaudible,” timestamp, and brackets.
        ii. For in-audible passages you can partially discern and/or aren’t confident, add any best-guess language into the brackets, leaving the “inaudible” word there, too.
        iii. For in-audible passages you cannot discern, just leave the “inaudible,” timestamp, and brackets.
7. Save the QC’d transcripts under “Transcripts Verified” with a new file name using a consistent naming convention.

Notes on De-identification:

Names of people should be replaced with the first letter of the person’s name (e.g., “Kate” would be transcribed as “[K]”)

Exact dates should be replaced with month and year only. For example, “1/21/2021” should be transcribed as “[January 2021]”.

Any email address, telephone number, fax number, or any other identifying information should be transcribed by naming the type of information identified in the audio recording (e.g., [Email], [Phone Number], etc.)

Clinician Interview Guide

READ: I'd like to begin our conversation about your experience working with the OBAT program through the COVID-19 pandemic. As a reminder, there are no right or wrong answers to any of the questions I will ask today, so please feel free to speak openly and honestly. Some of my questions deal with private and sensitive topics. You don't have to answer any questions that make you feel uncomfortable, and you can also stop the interview at any time. We are just interested in learning about your experiences and opinions. I would also like to audio record our conversation so I can focus on what you are saying without having to take too many notes. **Just to confirm, is it OK with you that I record this interview?**

Do you have any questions before we begin?

[TURN ON RECORDER; READ]: This is **[interviewer]** conducting a **clinician** interview with **[ID#]** on **[date]** at **[time]** via **[video/phone].**

SECTION 1: Opening and OBAT History up to COVID

1. **To get started, can you tell me about your role in the OBAT program?**
   a. **How many years have you been…**
      i. …working in this program?
      ii. …working professionally with patients with addiction?
   b. **Tell me about your key responsibilities and activities in a typical work day.**
   c. **When did you realize that COVID-19 was going to have an impact on your work?**

SECTION 2: COVID-19 Impact on OBAT

Now I'd like to ask you some questions about how COVID-19 impacted OBAT and your work. As a reminder, in mid-March, 2020, most non-COVID services went remote, including OBAT.

2. **From your perspective, what are the most important ways that COVID-19 impacted the <u>clinical services</u> provided through the OBAT program?**
   a. **How did OBAT transition to a remote model of care?**
      (Alternative wording/probe: What went into getting the clinic ready?)
      Probes:
      i. ***Telehealth and communication:***
         <u>For prescribers</u>: Tell me about the transition to telehealth due to COVID.
         <u>For nurses</u>: Tell me about the transition to telehealth <u>using cellphones</u>.
         <u>All</u>: How is it going now?
         What is it like to do intakes or new patient visits via telehealth only, never meeting a patient face-to-face, or limited witnessed medication inductions? (Pros/cons of this?)
         What was it like to run a 24-hour hotline?
         How could telehealth or communication with patients be improved?
      ii. ***Urine toxicology screenings:***
         What was it like eliminating urine toxicology screening due to the transition to remote care during COVID-19?

How is it going without any (or with fewer) urine toxicology screenings?
For new patients, what is it like to never having a single urine drug screen?

iii. ***Prescription length and access:***
What was it like increasing the length of prescriptions and numbers of refills due to COVID-19?
How is it going now?
How has COVID-19 impacted prescriptions for injectable naltrexone? Injectable buprenorphine?
How has it impacted administering injectable naltrexone or buprenorphine?
How do you think prescribing could be improved?

iv. For nurses: ***Frequency of contact with patients:*** What was it like having more frequent contacts or visits with patients following the start of the COVID-19 pandemic?

v. Any other changes as OBAT went remote care that I haven't mentioned?

b. How did COVID-19 impact your own work within OBAT?
Probes:

i. Did your job responsibilities increase or decrease?
ii. Tell me about any changes to your schedule due to COVID-19?
iii. Did you transition to working from home? If so, what was that like?
iv. How have your interactions with OBAT patients changed since operations at went remote due to COVID-19?
   - Changes in quality of communication/interactions via phone/text?
   - Changes in types of info/advice typically given to patients?
   - Do you think your relationship with patients has changed due to COVID-19? Why (not)?

3. How did COVID-19 impact your health and wellbeing?

a. Tell me about your experience with COVID-19 testing.
b. Have you had COVID-19 symptoms or illness?
c. Have you gotten the COVID-19 vaccine? Or will you? Why (not)?

4. How has COVID-19 impacted OBAT's patient population?

a. How has COVID-19 required OBAT to shift strategies to engage patients?
b. What has made it difficult for patients to…

i. …Keep up with appointments/stay engaged with OBAT?
ii. …Maintain adherence to their medication?
iii. …Access other recovery supports (e.g., in-person peer support; AA)?
iv. …Access to resources for other social determinants of health?
   - Mood, stress, mental health (and access to related services)
   - Housing
   - Domestic violence
   - Employment
   - Relationships with family, friends, other support persons
v. Access to other health services

c. What kinds of positive changes, if any, have you seen in patients' abilities to get or stay engaged with OBAT?

i. What types of patients benefit from telehealth/not having to be physically present?
ii. Are there other ways that some patients are doing well in this context?

d. What other risk factors related to COVID-19 are you concerned about for the OBAT patient population?
Probes:
i. Increase or changes in substance use; changes or contamination in the drug supply; overdose risk
ii. Changes in access to care due to clinics closing
iii. Access to treatment on demand for high-risk populations (e.g., post-incarceration)
iv. Accessibility issues for patients on injectable medications

SECTION 3: OBAT Suggestions and Closing

5. Reflecting on all of the changes made to the OBAT program during this pandemic, which changes would you recommend keeping? Why?
Probes:
a. Are there new changes due to COVID-19 that OBAT should adopt permanently?
b. Are there new changes due to COVID-19 that you'd like to see phased out or changed back?

6. What positive things have come out of this pandemic?
Probes: Positive changes for patients, for providers, for systems?

I believe those are all of my specific questions. Please give me just a moment to check to make sure I covered everything. **[CHECK OVER INTERVIEW GUIDE].**

7. Based on our discussion, is there anything else you think I should know?

This concludes the interview. **[TURN OFF TAPE RECORDER]** Thank you again for taking time out of your busy schedule to provide us with this information.

Clinician Codebook

Table of Contents

1. PROVIDER BACKGROUND INFO
2. IMPACT OF COVID ON PROVIDERS
2.1 Early Experience with COVID
2.2 Impact on Provider Workflow
2.3 Work from Home Experience
2.4 Provider Physical Health
2.5 Provider Mental Health
2.6 COVID Testing, Vaccination, Illness
3. CHANGES IN CARE PROCESSES
3.1 Intake
3.2 In-Person Visits
3.3 Telehealth Visits
3.3.1 Role of Technology
3.4 Patient-Provider Interactions
3.5 Quality of Care
3.6 Urine Toxicology
3.7 Prescriptions
3.8 Other OBAT Programs
3.8.1 24-Hour Hotline
4. IMPACT OF COVID ON PATIENTS
4.1 Medication Adherence
4.2 SDOH & Social Needs
4.3 Mental Health & Stress
4.4 Substance Use
4.5 Sexual Health
4.6 Access to Non-OBAT Health Services
4.7 Access to Recovery Supports
4.8 Diversion
4.9 COVID Testing, Vaccination, Illness
5. PATIENT ACCESS TO & ENGAGEMENT IN CARE
5.1 Role of the Environment
6. COSTS
7. LEADERSHIP
8. EQUITY
9. STIGMA
10. RACE/RACISM
11. GREAT QUOTES

REMINDER: Please code all of the content in the transcripts, excluding only content that is in no way relevant to the study (e.g., small talk).

Detailed Codebook

Name	Description	Notes/Example Quotes
**1. PROVIDER BACKGROUND INFO**	• Information about the interviewee's role, responsibilities, prior roles, time working in the OBAT clinic, time working in the field – generally this will come from the beginning of the interview<br>• Also apply this code if the interviewee self-identifies their race, ethnicity, or other aspects of identity in the interview	
**2. IMPACT OF COVID ON <u>PROVIDERS</u>**	• Any discussion of provider's personal experience with the impact of COVID that does not fall under the following sub-codes<br>• Include issues with family, e.g. their experiences with COVID, etc.	Example Quote: "He is an anxious kid. Like he came with me in the car behind [*hospital*] to pick up all my cell phones and all that stuff and he was panicking we were going to die of COVID, I'm like, 'You didn't even get out of the car.' But he had a lot of anxiety about that."
**2.1 Early Experience with COVID**	• Discussion of the interviewee's early experiences with COVID and realization of its impact on their work	
**2.2 Impact on Provider Workflow**	• Discussion of job responsibility and schedule changes (e.g. juggling new responsibilities, incorporating advance care planning, etc.)	
**2.3 Work from Home Experience**	• Discussion of positive and negative experiences with providers transitioning to working from home<br>• Discussion of work/life balance	Double-code with <u>Provider Physical Health</u> or <u>Provider Mental Health</u>, if applicable.
**2.4 Provider Physical Health**	• Discussion of how COVID impacted physical health, e.g. less exercise, effects of time spent on the computer, etc.<br>• This would include discussion of their experiences of physical ailments if the provider themselves got COVID	
**2.5 Provider Mental Health**	• Discussion of how COVID impacted mental health and well-being (both positive and negative impacts), e.g. feelings of isolation, stress<br>• This would include discussion of changes to their mental health if the provider themselves got COVID	
**2.6 COVID Testing, Vaccination, Illness**	• Discussion of providers' views, beliefs, attitudes regarding (and experiences with) COVID testing, vaccination, and illness	
**3. CHANGES IN CARE PROCESSES**	• Description of the structural changes to how the OBAT clinic was delivering care, e.g. transitioning prescribers to new visit formats, transitioning the method and frequency of nurse check-ins, staffing changes, distribution of phones to staff and patients, etc.	Example Quotes: "So, we always had somebody on-site to give injections and that was kind of like, our initial, we were having nurses there for injections. And so, it was a very skeleton crew, you know,

Name	Description	Notes/Example Quotes
	• Any discussion of the impact of COVID/transition to telehealth on care processes and clinical services that does not fall under the following sub-codes	one or two nurses every day, the rest of the staff are remote.”
**3.1 Intake**	• Description of the experience of intakes or new patient visits via telehealth and having limited witnessed medication inductions<br><br>*Additional Notes:*<br>Double-code with Patient Access to & Engagement in Care, if applicable.<br><br>Intake can be single-coded when a provider is discussing in-person intakes compared to telehealth; it does not need to be coded with either Telehealth or In-Person.	Example Quotes: “And then a patient who called 8am to our intake line saying, ‘I need help,’ they could talk to a physician within hours and get prescribed buprenorphine same day. So it was an effective way of getting people in pretty rapidly.”<br><br>“I think two years ago, we would have been like, ‘intake, without even meeting them, are you kidding me?’ But where the epidemic is, and people are dying, and just being able to offer a low barrier, you know, basically rapid access to care is just really important to us.”
**3.2 In-Person Visits**	• Discussion of positive aspects of in-person visits, e.g. being face-to-face, easier communication, more privacy, etc.<br>• Includes discussion of returning to in-person care for patients who require it, prefer it, etc.<br>• Discussion of negative aspects of in-person visits, e.g. patients having to travel, take time out of their days, etc.	Code *only* In-Person Visits when the discussion refers to care that can only be done in-person, e.g. urine toxicology, or is *primarily* discussion of in-person care.<br><br>Double-code with Telehealth Visits, if applicable, e.g. when provider is discussing how telehealth lacks the positive aspects of in-person care, such as being face-to-face, etc.
**3.3 Telehealth Visits**	• Discussion of provider experiences with providing care via telehealth, e.g. any difficulties they experienced, their preferences, etc.<br>• Discussion of positive aspects of telehealth visits, e.g. flexibility, ease of access for patients, etc.<br>• Discussion of negative aspects of telehealth visits, e.g. distractions for patients, less engagement from patients, etc.	
**3.3.1 Role of Technology**	• Discussion of technology issues on the provider end, issues on the patient end, lack of access to technology for patients, etc.	Does not need to be double-coded with Telehealth Visits. Role of Technology will be

Name	Description	Notes/Example Quotes
	• Includes discussion of patient's access to telephones and any mitigation strategies from the OBAT clinic	aggregated into the parent code, since they were being double-coded frequently, and technology tends to relate directly to telehealth visits. This is one of only two scenarios where coding is aggregated. Does NOT apply when parent codes have multiple child codes.
**3.4 Patient-Provider Interactions**	• Discussion of changes to the provider-patient relationship/bond/therapeutic alliance<br>• Discussion of changes to provider or patient goals in treatment interactions<br>• Discussion of changes to provider or patient tasks in treatment interactions, specifically referring to tasks occurring in the visit, e.g. more focus on certain aspects of the visit<br>o Example: Changes to interactions could include having to spend time during the encounter helping patients with technology, etc.<br>• Discussion of new mechanisms through which patients contact and interact with OBAT providers, including prescribers, nurses, recovery coaches, etc., e.g. via MyChart, texting, etc.<br>• Experiences of providers and perceived experience of patients with having more (or less) frequent contact with patients	Relevant probes from the interview guide:<br>- Do you think your relationship with patients has changed due to COVID? Why (not)?<br>- Changes in types of info/advice typically given to patients?<br><br>Double-code with Role of Technology, if applicable.
**3.5 Quality of Care**	• Discussion of any changes or differences in the quality of care based on the impact of COVID/transition to telehealth, e.g. differences in quality based on telehealth modality (phone vs. video visits)	[*Inductive Code*]<br>Relevant probe from the interview guide:<br>- Changes in quality of communication/interactions via phone/text?
**3.6 Urine Toxicology**	• Description of experiences with eliminating or reducing urine toxicology screenings<br>• Discussion of utility of urine toxicology screening, e.g. perceived risk of having less frequent screenings, the absence of objective clinical information, etc.<br>• Discussion of perceived benefits of less urine toxicology screening, e.g. improvements in patient-provider trust	
**3.7 Prescriptions**	• Description of experiences with changes to prescription access, prescription length, number of refills, etc.<br>• Includes discussion specific to prescriptions for injectable naltrexone and injectable buprenorphine, and administration of injectable naltrexone or buprenorphine in the context of COVID	

Name	Description	Notes/Example Quotes
**3.8 Other OBAT Programs**	• Discussion of COVID's impact on programs provided in the context of OBAT, e.g. PARC clinic for linking patients to care pre-release/post-incarceration, other programs for high-risk populations, etc.<br>• Discussion of any programs OBAT implemented in the context of COVID, e.g. support group via Zoom	[*Inductive Code*]<br>Example Quote: "Well, we have the, it's called the [name] clinic, and we had that before COVID… to go over to the jail and connect folks' prerelease, that kind of ended, they wouldn't let us in. But we still were the clinic that everyone got… released to. So [name], God love her, she was on seven days a week, they would call her when they were getting discharged and then I, Saturdays, I did that for a long time."
**3.8.1 24-Hour Hotline**	• Include any discussion of the provider's experience with the 24-hour hotline or discussion of their patients' experiences with the hotline	
**4. IMPACT OF COVID ON <u>PATIENTS</u>**	• Any discussion of the impact of COVID on patients that does not fall under the following sub-codes	
**4.1 Medication Adherence**	• Discussion of the impact of COVID on patients' medication adherence, e.g. changes in license requirements for picking up medication at the pharmacy<br>• This code should be used exclusively for discussion of medications, including barriers/facilitators to adherence as well as accessing prescriptions (e.g. picking up at the pharmacy)	Example Quote: "One thing that did help during the pandemic was the license requirements [were] relaxed, but that's back now. So, patients are struggling with that again."
**4.2 SDOH & Social Needs**	• Discussion of the impact of COVID on patients' ability to access housing, food, or employment; issues with domestic violence; and either positive or negative changes in relationships with family, friends, and other support persons<br>• Example: Can include discussion of how COVID (and its impacts, e.g. increased insecurity related to social needs, etc.) has led to competing priorities for patients	Example Quote: "I feel sometimes like doing these visits over the phone, um, I tend to-you know, patients tend to, sort of, brush me off, because they're, obviously, um, living through a pandemic and they have other things, I think, need to, sort of, um, address."
**4.3 Mental Health & Stress**	• Discussion of the impact of COVID on patients' mood, stress, mental health and well-being (both positive and negative impacts)<br>• Also includes discussion of patients' ability to access mental health services	
**4.4 Substance Use**	• Discussion of how COVID has led to changes in substance use behaviors (e.g. more risky injection behaviors, shifts to alcohol or cannabis, etc.)	

Name	Description	Notes/Example Quotes
	• Impact of COVID on patients' likelihood of relapse and/or overdose<br>• Impact of COVID on the drug supply, including changes or contamination	
**4.5 Sexual Health**	• Discussion of how COVID may have impacted and/or interacted with sexual health-related risk factors	[*Inductive Code*]<br>Example Quote: "For folks that were using or having unprotected sex, definitely the HIV, any other STDs… Often coincides with those risk factors as well. So any new people, and depending on what's going on in our lives, we definitely make sure we run labs for all of that. They retooled our questions and our visits. So we were added that sexual history component piece to our visits, not just in the intake, PrEP comes up now to offer to folks."
**4.6 Access to Non-OBAT Health Services**	• Discussion of the impact of COVID on patients' access to other health services (e.g. mental health care, specialty care, etc.)<br>• Impact of COVID on access to levels of care for addiction, e.g. inpatient detoxification, crisis stabilization programs, partial hospitalization programs, etc.	
**4.7 Access to Recovery Supports**	• Discussion of impact of COVID on access to recovery supports, including peer support, AA, NA, Smart Recovery, etc., both within and outside of the context of OBAT<br>• Includes discussion of impact on interactions with OBAT's recovery coach, e.g. the recovery coach spending time helping patients with technology<br>• Includes experiences of patients no longer accessing these services or shifting to virtual versions (e.g. patients' experiences attending AA/NA via virtual platforms)	This code could later be broken down into peer support within vs. outside of the context of OBAT.<br><br>Example Quote: "And then we have [name] to help folks download the group information onto their phones. So, some people they can just press that one button, and they get connected to the group. She's helped people get the meetings information on their phones, too."
**4.8 Diversion**	• Discussion of how COVID may impact diversion of prescription medication<br>• Include provider perceptions of diversion/risk of diversion in general<br>• Include provider perceptions of diversion in relation to care adaptations in the OBAT clinic (use of telehealth, less frequent visits, eliminating urine toxicology screenings, etc.)	

Name	Description	Notes/Example Quotes
**4.9 COVID Testing, Vaccination, Illness**	• Discussion of patients' views, beliefs, attitudes regarding (and experiences with) COVID testing, vaccination, and illness	
**5. PATIENT ACCESS TO & ENGAGEMENT IN CARE**	• Discussion of how COVID and/or the transition to telehealth may have affected access to care provided by the OBAT clinic (i.e. treatment initiation)<br>o Example: Discussion of low-barrier access to treatment provided by the OBAT clinic, e.g. requirements for entry are limited or minimal, treatment modality is based on patient preference, etc.<br>• Discussion of how COVID and/or the transition to telehealth may have affected engagement in care provided by the OBAT clinic (i.e. treatment retention)<br>o Example: Negative impacts of COVID on patients' ability to keep up with appointments and/or stay engaged with the OBAT clinic<br>o Example: Positive impacts of due to adaptations made by the OBAT clinic (e.g. use of telehealth, less frequent visits, etc.)<br>• Discussion of the types of patients that benefit from care adaptations (vs. those that do not)	[*Inductive Code*]<br>Double-code with Telehealth Visits, if applicable (e.g. in cases where discussion of engagement is linked to the use of telehealth, as this may represent a positive or negative aspect of telehealth encounters).
**5.1 Role of the Environment**	• Discussion of how the external environment/setting influences the way patients access/engage in care (either via telehealth or in-person), e.g. lack of privacy for visits, feelings of safety, etc.<br>• Discussion of issues such as transportation to the clinic, having to come to the Mass & Cass area (issues with triggers for patients), etc.<br><br>*Additional Notes:*<br>Does not need to be double-coded with Patient Access to & Engagement in Care. Role of the Environment will be aggregated into the parent code, since they were being double-coded frequently, and the environment tends to relate directly to patient engagement. This is one of only two scenarios where coding is aggregated. Does NOT apply when parent codes have multiple child codes.	Example Quote:<br>Interviewer: "What has made it difficult for patients to keep up with appointments?"<br>Interviewee: "I would say the biggest thing is fear for a lot of folks. Some are afraid, obviously just of [hospital]. Some, pre-COVID, were just afraid of the area, because I don't know if you walk around out there, but they offer you everything under the sun. And if they want to buy people's meds on top of that, there's a risk of violence all around there as well, that's happened to many of our patients, there's all that before COVID, that's still there."
**6. COSTS**	• Any discussion of finances/costs/reimbursement for services	[*Inductive Code*]
**7. LEADERSHIP**	• Any discussion of the role of leadership and management, including OBAT	[*Inductive Code*]

Name	Description	Notes/Example Quotes
	clinic/programmatic leadership, health system leadership, etc.<br>o Includes both administrative leadership and clinical leadership (e.g. leadership provided by nurses in the context of a nurse care manager model).<br>• Includes discussion of leadership priorities, e.g. competing priorities (patient vs. leadership preferences), etc.	Example Quote: "So is there anything, you know, that I could be missing that by prescribing these medications, I could be placing the-the patient in danger or increasing a risk for harm for the patient? I think [the clinic] really, uh, their number one, I think they were really on top of, uh, information as it was coming of like leading…like CDC and DEA and, you know, all these entities that are-that are sort of saying hey, this is okay to do…"
**8. EQUITY**	• Discussion of how COVID and/or the transition to telehealth may have affected equity in patients' care initiation, engagement, and/or outcomes	[*Inductive Code*]
**9. STIGMA**	• Discussion of providers' perspectives on stigma toward patients, etc.<br>• Providers' expressing stigma toward patients<br>• Discussion of patients' internalized stigma as a barrier to seeking help, etc.	[*Inductive Code*]
**10. RACE & RACISM**	• Discussion of provider experiences with race, racism, and racial justice movements concurrent with experience of COVID	[*Inductive Code*]
**11. GREAT QUOTES**	• Include any standout quotes that would be useful for manuscripts or grants; quotes that raise new or interesting topics that could warrant additional analysis or team discussion	Should be double-coded with other codes whenever possible; must be annotated with a memo re: significance and relationship to possible themes, key findings, or emergent topics.

Patient Interview Guide

READ: I'd like to begin our conversation about your experiences with the OBAT program. As a reminder, there are no right or wrong answers to any of the questions I will ask today, so please feel free to speak openly and honestly. Some of my questions deal with private and sensitive topics. You don't have to answer any questions that make you feel uncomfortable, and you can also stop the interview at any time. We are just interested in learning about your experiences and opinions. I would also like to confirm that it is OK for me to audio record our conversation so I can focus on what you are saying without having to take too many notes. **Just to confirm, is it OK with you that I record this interview?**

Do you have any questions before we begin?

[TURN ON RECORDER; READ]: This is **[interviewer]** conducting a **participant** interview with **[ID#]** on **[date]** at **[time]** via **[zoom/phone].**

SECTION 1: Opening and OBAT History

1. **To get started, can you tell me about how you started coming to this OBAT program?**
    - **a.** What was it like when you first got started with OBAT?
    - **b.** Why did you decide to enter treatment at OBAT?
    - **c.** How has your experience with OBAT changed over time?
    - **d.** Have there been times in the past when you got off of bupe/suboxone or stopped following up with OBAT?
    - **e.** Is there anything that motivated you to come to this OBAT program compared to a different clinic or program?

SECTION 2: COVID-19 Impact on OBAT Experience and Retention

In mid-March, 2020, most non-COVID health care services went remote.

2. **Tell me about your experience with remote OBAT visits using telehealth, either by video or phone.**
    - **a. What kinds of things have made it <u>difficult</u> for you stay involved in OBAT remotely?**
        - i. …Keep up with appointments / stay involved with OBAT?
        - ii. …Keep taking your meds every day?
        - iii. …Access other recovery supports (e.g., in-person peer support like from AA)?
    - **b. What kinds of things have <u>helped</u> you stay involved in OBAT remotely?**
        - i. …Keep up with appointments / stay involved with OBAT?
          (e.g., more frequent contact via texts, calls with nurses)
        - ii. …Keep taking your meds every day?
        - iii. …Access other recovery supports (e.g., recovery coach, peer support/AA)?

Now I'd like to ask you about your experience with some specific ways that OBAT operates that have changed since March, 2020.

3. **Tell me more about your experiences with <u>telehealth or remote visits</u>.**
    - **a.** What do you like about telehealth? What do you dislike?

i. ***For pre-COVID patients:*** How do you think telehealth compares to the traditional in-person model of OBAT?

b. Tell me about any experiences you've had with video calls.

c. What challenges have you experienced with technology (e.g., internet/phone access)?

4. **Tell me about your experiences with <u>urine drug testing</u>.**

a. Do you or did you find it helpful to do regular urine drug tests? Why (not)?

b. What has it been like to not be asked for urine drug tests (or be asked less often) in OBAT?

5. **Tell me about your experience <u>accessing buprenorphine</u>.**

a. Have you had any problems with:

ii. …The pharmacy?

iii. …ID requirements? (Or was this requirement suspended?)

iv. …Transportation? (Or, was this challenge reduced by telehealth?)

b. ***For pre-COVID patients:*** Have your buprenorphine prescriptions changed in length? What has that been like?

Now I'd like to ask you about your interactions with OBAT <u>prescribers like doctors or nurse practitioners</u> who you see every few months and who prescribe your medications.

6. **What is your communication like with OBAT prescribers?**

a. How often are you in contact with OBAT prescribers?

b. How do you prefer to stay in touch with OBAT prescribers (video, phone, texting)?

i. What kinds of things do you talk about on the phone? Or via text?

c. Have OBAT prescribers given you any advice about COVID-19? If so, what?

d. ***For pre-COVID patients:*** Do you feel that your relationships with OBAT prescribers has changed due to COVID-19? Why (not)?

Now I'd like to ask you about your interactions with OBAT <u>nurses</u>.

7. **What is your communication like with OBAT nurses?**

a. How often are you in contact with OBAT nurses?

b. How do you prefer to stay in touch with OBAT nurses (video, phone, texting)?

i. What kinds of things do you communicate about?

c. Have OBAT nurses given you any advice about COVID-19? If so, what?

d. ***For pre-COVID patients:*** Do you feel that your relationships with OBAT nurses has changed due to COVID-19? Why (not)?

8. **How COVID-19 has impacted your life?**

a. Do you feel that the pandemic has impacted your mood or mental health symptoms? (What about your level of stress? Have you felt lonely?)

i. If so, how are you managing this?

b. Is there anything about you or your situation, like your race, gender identity, religion, housing status, that has influenced how the pandemic has impacted you?

9. **How has COVID-19 impacted your recovery?**

a. We know that, in general, across the country, people have been drinking more during COVID-19, and in some places, drug-related overdoses are going up. For some

people aspects of COVID-19 has helped them avoid triggering situations or people. How much can you relate with these different experiences?

b. Can you tell me about any times when you started using drugs again?

10. **Have you received the COVID-19 vaccine? (If not) Will you get the COVID-19 vaccine? Why (not)?**
Probe: Is there anything about you or your situation, like your race, gender identity, religion, housing status that has impacted your willingness to be vaccinated?

OPTIONAL QUESTIONS (if time/not already discussed):

- How has COVID-19 impacted your:
  - …Housing?
  - …Employment?
  - …Relationships with family, friends, other support persons?
  - …Access to other health services?
- Tell me about your experiences with:
  - …COVID-19 testing
  - …COVID-19 symptoms or illness

SECTION 3: OBAT Suggestions and Closing

11. **Reflecting on your experience with OBAT during the COVID-19 pandemic, what suggestions would you have for improving the care/services you're receiving?**
a. Are there things about OBAT that you would like the OBAT clinicians to keep doing?
b. What advice would give to other OBAT patients? (e.g., how to stay engaged)

12. **Has anything positive come out of this pandemic?**

I believe those are all of my specific questions. Please give me just a moment to check to make sure I covered everything. **[CHECK OVER INTERVIEW GUIDE].**

13. **Based on our discussion, is there anything else you think I should know?**

This concludes the interview. **[TURN OFF TAPE RECORDER]** Thank you again for taking time out of your busy schedule to provide us with this information.

Patient Codebook

Table of Contents

1. PATIENT BACKGROUND INFO
2. EXPERIENCES WITH CARE*
**Do not use this parent code*
  2.1 Entering Treatment
  2.2 Disengagement from Care
  2.3 OBAT Experiences & Engagement
  2.4 Non-OBAT Experiences
3. RELATIONSHIPS WITH PROVIDERS
  3.1 Relationship with Prescribers
  3.2 Relationship with Nurses
4. IMPACT OF COVID-19 ON CARE PROCESSES
  4.1 Telehealth
    4.1.1 Technology
    4.1.2 Environment
  4.2 Urine Toxicology Screening
  4.3 Medication [Access]*
**Review code definition*
5. IMPACT OF COVID-19 (GENERAL)
5.1 Testing, Vaccination, Illness
5.2 Social Determinants
5.3 Relationships
5.4 Non-OBAT Health Services
5.5 Physical Health
5.6 Mood & Mental Health
5.7 COVID-19 & Recovery
5.8 COVID-19 & Peer Support
5.9 Positive Outcomes of the Pandemic
6. RECOVERY EXPERIENCES (OTHER)
7. SUGGESTIONS*
7.1 Suggestions to Other Patients
7.2 OBAT Process Improvements
**Review code definitions/notes (re: double-coding)*
8. GREAT QUOTES

REMINDER: Code all contents of each transcript, excluding only content in no way relevant to the study (e.g. small talk, technical issues, etc.)

Detailed Codebook

Name	Description	Notes/Example Quotes
**1. PATIENT BACKGROUND INFO**	• Discussion of the patients' personal and clinical background	Apply this code if the patient self-identifies their race, ethnicity, or other aspects of identity in the interview
**2. EXPERIENCES WITH CARE**	[*DO NOT USE PARENT CODE*]	
**2.1 Entering Treatment**	• Discussion of patient's decision to enter treatment at OBAT and early experiences with the clinic; this could be in response to questions and probes about what it was like to start OBAT (e.g., decision-making around treatment entry)<br>• Discussion of any factors that drew patients to [site] OBAT instead of another clinic (e.g., location, recommendation from a friend, etc.)	
**2.2 Disengagement from Care**	• Discussion of any instances where patient stopped following up with OBAT or stopped taking their medication either prior to or following onset of the COVID-19 pandemic	Related probe from the interview guide: Have there been times in the past when you got off of bupe/suboxone or stopped following up with OBAT?
**2.3 OBAT Experiences & Engagement**	• Discussion of general experiences with OBAT either prior to or following onset of the COVID-19 pandemic, including positive and negative aspects of the OBAT program<br>• Include discussion of negative impacts of COVID-19 on patients' ability engage in care (i.e., barriers related to staying engaged with OBAT)<br>• Include discussion of positive impact of COVID-19 on patients' ability to engage in care (i.e., facilitators related to staying engaged with OBAT)	Code can be applied when the patient replied in the negative, i.e. they had **never disengaged** from care, or when there is discussion of previous steady engagement with OBAT (with no prior periods of disengagement), e.g., "Interviewer: Have there been times in the past when you got off of Suboxone or stopped following up with OBAT? Interviewee: No."<br><br>Can include discussion of the general model/structure of OBAT, e.g., "Like, the only problem that I have with any of this, like opiate treatment, there is no, like-any plan. It's just kind of like, 'Okay, well, we'll see you next month.' I mean, versus, like, uh, a private or a paid clinic, they'll say, 'All right, we're gonna start you with this and gradually wean you down to nothing' … There's no-never, like, uh, a-a plan to get me off."<br><br>Can include discussion of the **absence** of any barriers related to COVID-19 and its impact on care engagement, e.g., "Interviewer: And are there things that you feel like made it more difficult to stay involved when you're doing visits remotely? Interviewee: Um, not necessarily."

Name	Description	Notes/Example Quotes
		When discussed in comparison with other service settings, can be double-coded with *2.4 Non-OBAT Experiences*
**2.4 Non-OBAT Experiences**	• Discussion of patient experiences with other SUD treatment services (e.g., MOUD treatment via non-[site] OBAT programs or methadone clinics, detoxification, partial hospitalization programs, etc.)<br>• Include patient experiences with Section 35 ("sectioning", "being sectioned"), i.e. being civilly committed and receiving involuntary SUD treatment	Example Quote: "I ended up actually at [hospital], I went… 12 times in a two-month period for, um, psych evaluations, overdoses, um, the, like, a-alcohol poisoning."
**3. RELATIONSHIPS WITH PROVIDERS**	• Any discussion of patients' relationship or interactions with OBAT staff/providers (e.g., doctors, nurse practitioners, nurses, or other OBAT care team members) that does not fall under the following sub-codes<br>• Discussion regarding the role of more frequent contact with OBAT providers/staff that occurred in the context of COVID-19 (e.g., check-in calls from nurses and OBAT recovery coaches)<br>• Discussion of patient preferences around contact frequency with the OBAT program/providers (in general)<br>• Discussion regarding the culture of the OBAT program/clinic and staff, e.g., patients' feeling that it is a supportive, empathetic, non-judgmental environment (or **not** feeling this way)<br>• Discussion of any ways that patients' relationships with providers has changed over time (e.g., trust being built)	In cases where the patient does not distinguish between prescribers and nurses (or other members of the care team, e.g., OBAT-affiliated recovery coaches) code data under the parent code, *5. Relationship with Providers*<br><br>In cases where the patient is comparing/contrasting or going back and forth between discussing prescribers and nurses, code data under the parent code, *5. Relationship with Providers*<br><br>Example Quotes: "It was, um, the clinic, the nurses and the doctors over there... I mean, because I talk to them about my feelings. I mean, I was always honest, you know, and I didn't hide anything. And-and-and-and you know, they were still so supportive. They didn't judge me. They didn't… You know, they understood and they gave me a little bit more support. You know, a little bit more motivation. And so, uh, that's what kept me. And if-if they keep believing in me and supporting me, then I gotta keep believing in myself and supporting myself."<br><br>"I got to know a lot of them better. Uh, the calls felt a lot more personal. Uh, some were very open to talking and, you know, talking about their lives and asking about mine. So, we probably had in-more in-depth conversations that I-I would have had in person visits."
**3.1 Relationship with Prescribers**	• Discussion of patients' relationship or interactions with OBAT prescribers (i.e., doctors or nurse practitioners who patients see every few months and who prescribe their medications)	

Name	Description	Notes/Example Quotes
	• Include patient preferences around contact frequency with prescribers, specifically<br>• Include any description of advice that prescribers gave to patients about COVID-19	
**3.2 Relationship with Nurses**	• Discussion of patients' relationship or interactions with OBAT nurses<br>• Include patient preferences around contact frequency with nurses, specifically<br>• Include any description of advice that nurses gave to patients about COVID-19	
**4. IMPACT OF COVID-19 ON OBAT CARE PROCESSES**	• Any description of the impact of COVID-19 on OBAT care processes that does not fall under the following sub-codes	
**4.1 Telehealth**	• Discussion of patient experiences with remote (phone or video) visits with OBAT<br>• Include discussion of patient preferences method of communication with providers (e.g., in-person vs. telehealth)<br>• Include discussion of patient perspectives about how telehealth compares to in-person visits<br>• Include discussion of the content of any conversations with OBAT providers via telehealth	Example Quote: "Because like if you're in person you-you actually sittin' down with a real person. Not saying that you're not talking to somebody real on the phone, but what I'm saying is, when you in-when you in person, I mean, you-you're given direct contact, really trying to explain face to face with that person. Um, you have the eye contact with that person and you can really focus on how somebody, uh, presents their self like, uh, fidgety, nervousness, uh, twiddling your thumbs, or just like the an-anticipation of-of trying to-trying to really get the point across what the problem is in person instead of just like okay, okay, is this phone call gonna end."<br><br>Can include discussion of patients' ideal balance between telehealth and in-person care, e.g., "I would say, via phone and video for the majority of time, but every, um, you know, six months, uh, going in for a visit, um, is absolutely necessary, I guess for other health reasons."
**4.1.1 Technology**	• Discussion of any challenges patients have experienced using technology to access telehealth visits<br>• Responses to questions or probes about patient challenges with using Zoom or having inconsistent phone or internet access	
**4.1.2 Environment**	• Discussion of how patients' environment impacts their engagement with telehealth (e.g., lack of privacy due to	Example Quote: "Now that I think about that question a little bit more, yes. Because I had the telephone visits there was a time when it was even worse, um, like in the [clinic] area

Name	Description	Notes/Example Quotes
	living with others or staying in shelter, etc.)<br>• [**Conversely**] Discussion of how telehealth implementation may have impacted patients' experiences with the environment (e.g., not having to go into the clinic, not having to go to the Mass & Cass area, etc.)	and being able to not have to go down there, especially in the summer months was very, very helpful."
**4.2 Urine Toxicology Screening**	• Discussion on patients' attitudes towards urine toxicology screening pre-pandemic and any changes in attitudes over time<br>• Discussion of patients' experiences with reduced urine toxicology screenings during the pandemic	
**4.3 Medication [Access]**	• Discussion of patients' experiences with MOUD (e.g., Suboxone, Sublocade, Vivitrol, etc.) in general<br>• Discussion of patient experiences with accessing and adhering to their MOUD during the COVID-19 pandemic (e.g., changes to ID requirements, issues with transportation, problems at the pharmacy, etc.)<br>• Include discussion about any changes to the length of patients' prescriptions	This code should be considered *4.3 Medication,* though it will not be changed in NVivo. Note that it can be applied more broadly (not just in discussion of access to medication).<br><br>Code can be applied when there were concerns about the impact of COVID, or potential impacts, that did **not** occur, e.g., "That was one of the things I was a little concerned about, um, especially being on a medication like Suboxone. Um, which is-you can have severe withdrawals, um, it's not a medication that you wanna go without."
**5. IMPACT OF COVID-19 ON PATIENTS (GENERAL)**	• Any discussion of the impact of the COVID-19 pandemic on patients that does not fall under the following sub-codes<br>• Include discussion of how a patient's identity/circumstances may have influenced (or did **not** influence) how COVID-19 impacted them	Related probe from the interview guide: Is there anything about you or your situation, like your race, gender identity, religion, housing status, that has influenced how the pandemic has impacted you?
**5.1 Testing, Vaccination, Illness**	• Discussion of any patient experiences with being tested for COVID-19<br>• Discussion of patient/partner/family experiences with having symptoms of COVID-19 or being diagnosed with COVID-19	Example Quote: "So, [my mom] got the shot. She got the shot early. My uncle, he's old. He got the shots. So, I stay with my mom. So, they both have had it. So, I'm not worried about getting it now, even though they say now you can. That was the big reason why. And also, number two, I don't believe the – uh, okay. So, my theory is that, um – my theory is that we are at a level that should be herd immunity."

Name	Description	Notes/Example Quotes
	• Discussion of any experiences with work/community exposures<br>• Discussion of patient experiences with deciding whether or not to receive a COVID-19 vaccination, and their experiences with receiving a vaccination, if applicable<br>• Include discussion of any aspects of patients' identity (gender, race, religion, housing status, etc.) that impacted their willingness to be vaccinated	
**5.2 Social Determinants**	• Discussion of how COVID-19 has impacted patients' housing, employment, food access, finances, or other social determinants of health	Example Quote: "I'm in a sober house right now because it's getting paid for by a grant, other than that I'd be homeless, you know what I mean, 'cause I lost my job and then I wasn't able to pay my rent, you know, I… The family members I have have limited income themselves, so um, and they're, you know, they've got their own issues, so. But, yeah, I feel that it definitely affected my housing."
**5.3 Relationships**	• Discussion of how COVID-19 has impacted patients' personal relationships outside of OBAT (e.g., with friends, family, partner/spouse, etc.)	Example Quote: "I mean, it-it's-it isolated me from my family, so it was kind of-of a different-different, um, different scenario for me. It was, where I couldn't see them, I know for some people, like, their family can be triggering, so maybe that's a situation, but for me, it-it put a block with me. And I was like, what the heck am I doing clean and sober if I can't see my loved ones."
**5.4 Non-OBAT Health Services**	• Discussion of how COVID-19 has impacted patients' access to other health services (e.g., other SUD treatment services, mental health care, physical health care, etc.)	Example Quote: "I couldn't go see my counselor anymore at [another clinic], I was going there to see my psychiatrist."
**5.5 Physical Health**	• Discussion of how COVID-19 impacted patients' physical health and comorbidities	
**5.6 Mood/Mental Health**	• Discussion of how COVID-19 has impacted patients' mood or mental health<br>• Discussion of patients' strategies for managing/coping with negative mood/mental health, e.g., therapy, friends, faith, etc.<br>• Include discussion of fear/anxiety regarding getting COVID-19, social distancing, etc.	Example Quote: "But I am terrified, I don't want-I know people get it again. I do not want to get it again."

Name	Description	Notes/Example Quotes
**5.7 COVID-19 & Recovery**	• Discussion of how COVID-19 has impacted patients' recovery, including how the pandemic may have helped or hindered it (e.g., avoiding triggering situations or people, etc.)<br>• Discussion of how COVID-19 impacted patients' substance use behaviors (e.g., use of different substances, increased drinking/smoking, polysubstance use, risk of overdose, etc.)<br>• Include discussion of relapse in the context of COVID-19	Example Quote: "I did have a lapse. A lapse is when you use once, or even twice but then you continue to stay focused and keep doing what you've been doing. A relapse is when you relapse and you're-now you're back into continuously consistently using drugs, you know, daily, regularly. So I didn't relapse but I did have a moment of weakness, you know, and I d-and I-and I did use. I did. But I think it-it-it helped me because it scared the heck out of me and, uh, it made me realize that that's what waiting there for me. If I relapse again, if I use again, that's what's waiting there for me. And I don't want that. So it was just a reminder. It was an eye-opener. It was a slap upside my head to make me see and realize that that disease is right there waiting for you-for you to mess up. And so it reminded me of where I didn't want to go back to."
**5.8 COVID-19 & Peer Support**	• Discussion of the impact of COVID-19 on patients' access to and experience with peer recovery support (e.g., AA/NA)<br>• Include discussion about technology, relationships, etc. in relation to patients' experiences with peer recovery support	Example Quote: "It's already hard to, you know, get up and share about whatever we're going through in our individual recovery processes so to have it be via, you know, remotely is-it's difficult."<br><br>When the impact of COVID-19 on access to other recovery supports (e.g., AA/NA) is discussed in relation to patients' ability to stay engaged with OBAT, double code with either *3.1 Barriers to Staying Engaged* or *3.2 Facilitators to Staying Engaged*, as applicable
**5.9 Positive Outcomes of the Pandemic**	• Discussion of any positive outcomes of the COVID-19 pandemic (those **not** related to OBAT care experiences) (e.g., more time spent with family, increased resiliency, etc.)	Related question from the interview guide: Has anything positive come out of this pandemic?
**6. RECOVERY EXPERIENCES (OTHER)**	• Any discussion of a patient's experiences with recovery that does not fall under other codes (e.g., *5.7 COVID-19 & Recovery* [which focuses on how COVID specifically impacted their recovery], *5.8 COVID-19 & Peer Support*, *2.3 OBAT Experiences/Engagement*, etc.)<br>• Include discussion of a patient's relationship/experience with forms of peer recovery support (e.g., AA/NA, other forms of peer support) that is broader than how COVID-19 impacted their experience<br>• Include discussion of influence of social determinants of health	[*Inductive Code*]<br><br>Example Quote: "And when you go to AA, you hear how this turned to this and that, but what – I mean, I can't say for everybody – but for me it was just like NA, NA was just like how you use. It was a big trigger for me because here, you know, here you have somebody, um, explaining how they did it, uh, it's oh, I just shot a gram of dope."<br><br>Can include patient's perspectives on others' recovery experiences, e.g., "I know other people that just skate – like they skate around it, like I told you. They don't take drug tests, or they're getting high and then they just get their medicine, and then they do whatever, you know. That's – it's what they do, you know. I don't know. I don't know. It is what it is, I

Name	Description	Notes/Example Quotes
	on patient's recovery (e.g., finances, housing, etc.) that is broader than the impacts of COVID-19	guess. That's the way life works, and – but it's not good. It's not help – it's not – it's not helping nobody. Well, no, it's not helping those people."
**7. SUGGESTIONS**	• Any discussion of patients' suggestions or advice that does not fall under the following sub-codes	Related question from the interview guide: Reflecting on your experience with OBAT during the COVID-19 pandemic, what suggestions would you have for improving the care/services you're receiving?
**7.1 Suggestions to Other Patients**	• Discussion of any suggestions or advice that patients would give to other OBAT patients (e.g., how to stay engaged)	*This will often be double-coded with content codes above.*<br><br>Related probe from the interview guide: What advice would give to other OBAT patients? (e.g., how to stay engaged)<br><br>Responses to the interview question that relate to content areas can be double-coded as applicable, e.g., with *3.2 Relationship with Nurses* – "Interviewer: And what advice would you give to other OBAT patients? So, like any advice on how to stay engaged or – or anything else that you think would be important to know? Interviewee: I would suggest maybe like getting a nurse that you trust and like. Like I trust my nurse. Like I told you, I can tell her anything."
**7.2 OBAT Process Improvements**	• Discussion of any suggestions or advice patients would give for how to improve the care or services that OBAT offers<br>• This sub-code could also include any content that is relevant to improving care quality/processes, even if the patient is not making a direct suggestion	*This will often be double-coded with content codes above.*<br><br>Related probe from the interview guide: Are there things about OBAT that you would like the OBAT clinicians to keep doing?<br><br>Responses to the interview question that relate to content areas can be double-coded as applicable, e.g., with *4.2 Urine Toxicology Screening* – "Interviewer: Do you find it helpful to do the regular urine drug tests, or do you not? Interviewee: No. I found it extremely expensive to the point where I'm not even positive if I can continue going. Because I've somehow racked up like over a thousand dollars medical bills and I have the insurance. And it's because of the drug tests."<br><br>Example Quote: "I wish that there was like a, uh, I don't-I don't know, like, I don't know what you would call them. Like, a recovery, like, uh, coordinator. Somebody that says, 'Okay, you're started on this date in 2022, we're gonna try to get you out and-and completely, uh, medication-free by 2025.'"
**8. GREAT QUOTES**	• Include any standout quotes that would be useful for manuscripts or grants; quotes	Should be double-coded with other codes whenever possible; must be annotated with a memo re: significance and relationship to

Name	Description	Notes/Example Quotes
	that raise new or interesting topics that could warrant additional analysis or team discussion	possible themes, key findings, or emergent topics
